# Perception and adaptation of pastoralists to climate variability and change in Morocco's arid rangelands

**DOI:** 10.1016/j.heliyon.2021.e08434

**Published:** 2021-11-23

**Authors:** Wadii Snaibi, Abdelhamid Mezrhab, Oumar Sy, John F. Morton

**Affiliations:** a National Institute of Agricultural Research, CRRAO, Oujda, Morocco; b Laboratory Communication, Education, Digital Usage and Creativity, ETIGGE Research Team, Mohammed Premier University, Oujda, Morocco; c Geomatics and Environment Laboratory, Assane Seck University, Ziguinchor, Senegal; d Natural Resources Institute, University of Greenwich, London, United Kingdom

**Keywords:** Climate change, Livestock production, Vulnerability, Adaptation, Determinant, Binary logistic regression

## Abstract

Since the late 1970s, extensive livestock production in the high plateaus of Eastern Morocco, particularly of small ruminants, has been seriously threatened by climate change (CC). Negative impacts include reduction in rangeland forage production and water availability, increased poverty and inequality, and increased degradation of rangelands. Different categories of pastoralists have adopted different combinations of adaptation strategies, but the factors influencing adoption have not to date been investigated. This paper aims to identify the perceptions of pastoralists on CC, to analyze the adaptive responses of different wealth categories, and to determine the factors affecting the adoption of adaptation measures. The Mann-Kendall, Pettitt and Buishand tests and the standardized precipitation index were used to analyze the climate data. Data on adaptation were examined using the chi-square homogeneity test, Kruskal-Wallis test and binary logistic regression.

The observed climate trends perfectly corroborated pastoralists' perceptions of significant changes in their local climate since the 1970s: a considerable decrease in annual rainfall and an increase in temperature and frequency of droughts and high winds. There were significant differences (Chi square = 7.603, *p* = 0.022, df = 2) between small, medium and large pastoralists in the frequency adoption of adaptation strategies, especially between small and large pastoralists (U statistic = 16.000, *p* = 0.009). The distribution of most adaptation actions also differed significantly between these two groups. Wealthier pastoralists have adopted a greater range of strategies, while poorer pastoralists have less diverse adaptation portfolios, and are more likely to adopt less advantageous strategies such as casual labor. The adoption of adaptation practices was significantly influenced by equipment, educational level, household size, herd size, training received, CC perceptions and agroecological setting. Public interventions to improve the adaptive capacity of pastoralists in Morocco's arid rangelands should be geared towards addressing these determinants and should prioritise small-scale pastoralists.

## Highlights

• Pastoralists' perceptions of climate change accord with observed climate trends.
• The nature and number of pastoralists' adaptive responses vary by wealth.
• Poor herders are more likely to adopt problematic strategies such as casual labor.
• Pastoralists' adaptive responses are specific to local agro-ecological conditions.
• Perceptions, adaptations and socioeconomics are significantly correlated.

## 1 Introduction

Livestock farming is a major component of Moroccan agriculture, ensuring different economic, social and nutritional roles. It contributes up to 30% to the agricultural Gross Domestic Product, provides about 20% of employment in rural areas and guarantees the food security of the country in red meat (MAPMDREF, 2014). The national herd structure is largely dominated by small ruminants (88%) with 19.2 and 6.2 million head of sheep and goats respectively (MAPMDREF, 2019). In the high plateaus of eastern Morocco (HPEM), which represent one of the country's most extensive pastoral areas, the importance of rearing small ruminants, mainly sheep, is even more obvious, fulfilling economic, nutritional, sociocultural and environmental functions. Small ruminant production is the main economic activity and job provider for local households (Bechchari et al., 2014; Snaibi, 2020a), it is a major source of red meat supply, provides cash that can be easily mobilized when needed and constitutes a significant cultural value which gives larger producers a high social status. Due to its heavy reliance upon natural resources and hence on climate conditions, extensive livestock production is particularly vulnerable to climate change (CC) (Bechchari et al., 2014; Herrero et al., 2016; Menghistu et al., 2020). Indeed, this economic activity is practiced in an arid pastoral ecosystem which exhibits important water stress and a climate characterized by low and highly variable rainfall combined with frequent droughts (Bechchari et al., 2014; Melhaoui et al., 2018; Snaibi, 2020b). Since the mid-1970s, the study area has shown obvious manifestations of CC giving rise to warmer and drier weather conditions (Born et al., 2008). Indeed, the precipitation has registered a considerable and widespread decrease (Fink et al., 2010; François et al., 2016; Melhaoui et al., 2018; Snaibi, 2020b), while the temperature and the frequency of droughts have increased (Moroccan Meteorological Office, 2007; François et al., 2016; Melhaoui et al., 2018). These unfavorable trends in rainfall and temperature regimes could continue for decades to come (Born et al., 2008; Mokssit, 2012; Schilling et al., 2012; Maroc, 2016; Meddi and Eslamian, 2021).

Consequently, CC has affected human and natural environments of the study area giving rise to manifold effects. These include a fall in forage production from rangelands and amplified water stress, leading to a scarcity of fodder and water resources (Mahyou et al., 2010; Maâtougui et al., 2011), which in turn increases competition over existing pastoral resources (Bourbouze and El Aich, 2000). In addition, climate change and extremes (droughts) are one of the main factors responsible for rangeland degradation in the high plateaus of eastern Morocco, which has also been generated by overgrazing, clearing and conversion of rangelands into cultivated land as well as an inefficient pasture management (Mahyou et al., 2010; Maâtougui et al., 2011; Schilling et al., 2012; Bechchari et al., 2014; Lang et al., 2021). Furthermore, extreme weather events induced by CC have increased poverty among poor small-scale pastoralists and rural-urban migration (El Harizi et al., 2005) and exacerbated social inequalities among pastoralists, especially during persistent droughts (Schilling et al., 2012). Thus, in severe droughts, for instance, the vulnerability of small-scale pastoralists is heightened, as they are unable to buy higher-priced livestock feeds and they face increased competition on available fodder resources. Therefore, they are forced to sell part of their herds to feed the rest of the livestock and provide for their families, engage in temporary wage labor or even give up livestock rearing and leave for neighboring cities in search of work (Bourbouze, 2000; Bechchari et al., 2014). After severe or prolonged droughts, it is very difficult for small-scale pastoralists to reconstitute their herds. Conversely, large-scale pastoralists are relatively less dependent on natural resources for the formation of their income, have the financial means to buy animal feed even in times of crisis, and above all they can reconstitute their herds once the climatic conditions become more favorable or after the advent of subsidized feed (Kuhn et al., 2010; Bechchari et al., 2014). Hence, the poorest pastoralists with livestock -based livelihoods are hardest hit owing to their increased vulnerability to the adverse consequences of climate change and extremes (Snaibi, 2020b). Given all these multiple impacts induced by CC, the sustainability of extensive livestock farming (based on grazing) and of the entire pastoral ecosystem is increasingly compromised.

To face or mitigate these adverse impacts regarding climate variability and change, pastoralists in the study area have implemented a varied set of coping and adaptation practices. These include rearing of mixed herds of sheep and goats, integration of crop and livestock production, sale of livestock to buy animal feed, pastoral mobility, destocking, storage of livestock feed, commercializing of livestock, migration, climate insurance, diversification of income or livelihoods, use of emigrants' remittances, social networks and intra-community solidarity to mitigate income shocks and using subsidized livestock feed (Bourbouze, 2000; Bourbouze and El Aich, 2000; Schilling et al., 2012; Bechchari et al., 2014; Snaibi, 2020a). However, pastoralists have adopted these coping and adaptation strategies differentially. Indeed, their behavior and adaptive abilities depend on their respective socio-economic conditions (Bechchari et al., 2014). Hence, small-scale pastoralists only have the opportunity to implement a limited number of adaptation measures, thus exhibiting lack of diversity in their adaptation portfolios, and they are more exposed to the risk of abandoning livestock production particularly in the event of recurrent and persistent extreme weather events such the droughts (Bourbouze, 2000; Bechchari et al., 2014; Snaibi, 2020b). Moreover, several public interventions have been implemented to support pastoralists in their efforts to manage the negative consequences of extreme weather events (in particular drought) and CC. These are the provision of subsidized livestock feed, the mobilization of water for livestock watering and insurance against multiple climatic risks. However, the fodder subsidy policy has generated some counterproductive effects such as maintaining or even increasing the number of livestock in spite of droughts, thus preventing the natural and usual reduction of grazing pressure on rangelands in such periods of crisis, and therefore overgrazing and sedentarization have increased (Schilling et al., 2012; Freier et al., 2014). Since the available climate insurance compensation covers only losses incurred in land cultivated with cereals and not livestock (sedentary or mobile), this instrument has sparked a race to clear rangelands in order to convert them into cultivated lands. In addition, these public measures have been of greater benefit to wealthy pastoralists with instant financial resources.

In addition, perception that climate change is happening is the most important step before adaptation (Gbetibouo, 2009) or a prerequisite for it (O 'Brien et al., 2006; Thomas et al., 2007; Silvestri et al., 2012). In fact, the farmers' perception of long-term changes in their usual climatic conditions, influences both their decisions to adapt or not (Deressa et al., 2009; De Longueville et al., 2020), as well as their choices of adaptive responses to be implemented (De Longueville et al., 2020). Also, the perception of CC is a key factor conditioning the success of the adaptation measures and strategies to be put in place (Kemausuor et al., 2011; Tesfahunegn et al., 2016). Accordingly, a deeper understanding of farmers or pastoralists' perceptions of CC is useful in developing relevant adaptation programs and policies to reduce the negative impacts and vulnerability induced by CC (Speranza, 2010; Bryan et al., 2013; Zampaligré et al., 2014; Van Wesenbeeck et al., 2016), especially in arid ecosystems (Opiyo et al., 2015; Kosmowski et al., 2016; Nnko et al., 2021).

The literature concerning adaptation to CC in agricultural systems indicates that adaptation is influenced by a set of socio-economic, institutional, sociocultural, geographic and perceptual factors (Dasgupta et al., 2014; Opiyo et al., 2015; Idrissou et al., 2020; Snaibi, 2020a; Nnko et al., 2021). These include the socioeconomic characteristics of farmers such as age, education level, family size, livestock size; institutional factors like credit access, training and extension services; agroecological settings (location) as well as perceptual climate variables such as perceptions of long-term changes in rainfall and temperature.

Considering all of the above, this study looks at filling the knowledge gaps around differentiation by wealth and other socio-economic variables in the adoption of CC adaptation practices by pastoralists. It also aims to help pastoralists and policy-makers to put in place effective and appropriate adaptation measures toward climate variability and change. In addition, the identification of factors influencing the adaptation strategies of pastoralists at the local level makes it possible to identify the levers likely to improve or strengthen this adaptation in the face of CC. In order to take into account the existing differences in local socioeconomic and biophysical conditions, we favored local-level analyses as suggested by several previous studies such Debalke (2011), Below et al. (2012), Obayelu et al. (2014) and Belay et al. (2017). The study therefore aims to answer three research questions. First, we will examine what are pastoralists' perceptions with regard to CC and whether they are consistent with the climate trends actually observed. Second, we will look whether there is a difference between socio-economic categories of pastoralists in adapting to perceived changes in local climatic conditions. Finally, we will analyze what are the key factors that influence the pastoralists’ decision in adopting particular adaptation strategies in response to CC. This study is based on the following two hypotheses:

- Different socioeconomic, institutional, geographic and perception factors influence the abilities of pastoralists to adapt to climate change.
- The larger the herd of a pastoralist, the higher the frequency of adoption of adaptation practices and their diversity.

## 2 Methodology

### 2.1 Study area

**Figure 1:**
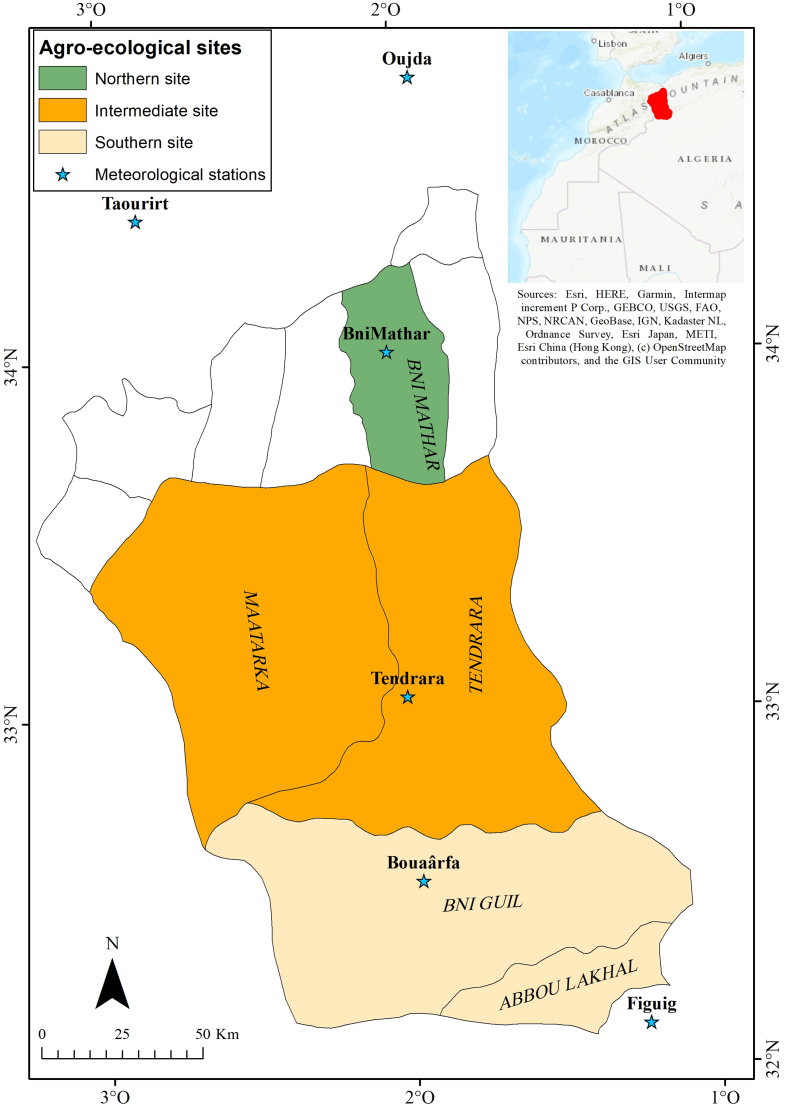
Location map of the study area.

This study was carried out in the high plateaus of eastern Morocco (HPEM), which lie between latitudes 32°00′N and 34°00′N and longitudes 1°00′W and 3°00′W, covering an area of about 3.5 million hectares, with an altitude varying from 900 to 1,400 m above sea level (Figure 1). Their soils are silty to sandy-silty, formed by quaternary deposits and the tertiary limestone substrate, with a great tendency to crusting (Bechchari et al., 2014). Their organic matter content is very low (less than 1%) and they are sensitive to wind and water erosion (Mahyou et al., 2016). Available water resources are very restricted. The study area is characterized by a semi-arid to arid bioclimate in the north and hyper-arid or pre-Saharan bioclimate in the southern part (Table 1). In fact, there is a decreasing rainfall gradient from north to south, with respective average precipitations of 215 and 146 mm (Snaibi, 2020b). The rainfall exhibits a high interannual variability, with a coefficient of variation ranging between 34% and 45% (Melhaoui et al., 2018). The annual temperature is on average 19 °C, but shows a high amplitude (Ben El Mostafa et al., 2001). In summer, hot and dry winds that can lead to strong sandstorms, are frequent. The vegetation consists mainly of steppes of *Stipa tenacissima*, *Artemisia herba-alba* and Chenopodiaceae (*Artrophytum scoparium*). The forage productivity of rangelands is generally low (Mahyou et al., 2018) and due to increasing pressure on natural resources, almost half of the rangelands in the study area suffered severe degradation (Mahyou et al., 2016). The population is about 80,000 inhabitants, for whom pasture-based livestock production, mainly of sheep, is the main source of livelihood. From the end of the 1970s, the high plateaus of eastern Morocco showed proven signs of climate change, namely a considerable decrease in annual rains combined with an increase in temperature and frequency and intensity of droughts (Jorion, 2009; Mahyou et al., 2010; Maâtougui et al., 2011; François et al., 2016; Melhaoui et al., 2018). Therefore, the study area represents an appropriate and representative pilot site for a better investigation and understanding of the perceptions and adaptation of pastoralists with regard to climate variability and change in the pastoral ecosystems of Morocco.

**Table 1:** Differences in socioeconomic and biophysical features between the three study sites.

Agroecological sites	North	Intermediate	South
Population (number of inhabitants)	8,869	14,869	8,743
Density of population (inhabitants/Km^2^)	High	Low	Low
Area (Km^2^)	1,793	17,359	11,806
Arable land (ha)	23,582	9,495	13,168
Irrigated area (ha/household)	1.76	0.1	0.6
Maximum altitude (m)	1,000	1,668	1,839
Aridity index^1^	Upper	Middle	Lower
Climate	Semi-arid to arid	Arid	Arid to hyperarid
Mean rainfall (mm)	215	199	146
Coefficient of variation of rainfall (%)	34	47	44
Mean temperature (°C)	16.4	17	20
Availability of irrigation water	Relatively high	Very limited	Very limited
Extent of rangelands	Reduced	Great	Medium
Type of livestock system	Semi-intensive	Semi-extensive to extensive	Semi-extensive to extensive
Sheep numbers (heads/household)	57	154	110
Goat numbers (heads/household)	9	25	31
Main livelihoods	Rearing of SR^2^ and cattle & LIA^3^	SR rearing	SR rearing and wage work

Note: ^1^Aridity index (AI): Upper arid (0.21 ≤ AI ≤0.26), Middle arid (0.15 ≤ AI ≤0.21), Lower arid (0.09 ≤ AI ≤0.15); ^2^SR: Small ruminants; ^3^LIA: Localized irrigated agriculture.

### 2.2 Site selection and sampling methods

A multi-step sampling technique was employed to select study sites and sample heads of livestock-producing households (hereafter referred to as “pastoralists”) in the study area. In the first stage, three agroecological sites were selected using a purposive sampling method due to their contrasting biophysical and socioeconomic conditions, which indicate differentiated agroecological environments. The zones studied include the northern agroecological site (rural territorial collectivity of Bni Mathar), the intermediate agroecological site (rural territorial collectivities of Tendrara and Maâtarka) and the southern agroecological site (rural territorial collectivities of Bni Guil and Abbou Lakhal). This selection of study sites was based on a set of criteria, mainly: climatic conditions, irrigation water potential, altitude, availability of large expanses of natural pastures, type of predominant livestock farming system and diversification of sources of income or main livelihoods (Table 1). Given the contrasting agroecological conditions, the north part of the study area shows relatively high agricultural potential, while the southern zone represents an area with low agricultural potentialities.

The second stage consisted of randomly selecting 167 pastoralists in proportion to the total number belonging to each selected study site (Table 2). In the final stage, in order to capture the major variations in the adaptation strategies of livestock producers, three wealth categories of pastoralists were identified, depending on the size of the herd of sheep held, following consultation with local development and extension agents. A large-scale pastoralist is the one who owns a flock of more than 300 heads of sheep, a medium pastoralist possesses a sheep herd ranging from 101 to 300 heads and a small-scale pastoralist owns a sheep herd not exceeding 100 heads. Thus, taking into account the relative representativeness of the three categories of pastoralists (small, medium, large) in each site studied, the pastoralists interviewed were sampled at random. The sampled pastoralists, who total 167, are distributed as follows: 96 small-scale pastoralists, 47 medium and 24 large-scale pastoralists.

**Table 2:** Distribution of pastoralists interviewed according to study sites.

Agroecological sites	Number of rural territorial collectivities	Number of rural territorial collectivities selected	Total number of households^1^	Sample size	Number of pastoralists surveyed by category^2^
Northern site	7	1	1,469	30	14S, 12M, 4L
Intermediate site	2	2	2,502	82	50S, 19M, 13L
Southern site	2	2	1,595	55	32S, 16M, 7L
Total	11	5	5,566	167	96S, 47M, 24L

Note: ^1^ Haut-Commissariat au Plan. (2015). *General census of population and housing, 2014*. *Legal population of regions, provinces, prefectures, communes, districts and communes of the Kingdom of Morocco*, [Table]. Retrieved from https://www.hcp.ma/downloads/RGPH-2014_t17441.html; ^2^ S: Small, M: Medium, L: Large.

### 2.3 Data collection

Study data were gathered through a survey of pastoralists heads of households, carried out between September and December in 2015. A semi-structured questionnaire was used to collect the necessary information on the socio-economic characteristics of pastoralists, their perceptions of the main changes in climate and the frequent climatic hazards that the study area has experienced over the past five decades, access to institutional services (credit, training, organization) as well as the adaptation and coping practices they have undertaken in response to the negative impacts of climate variability and change. Historical climate data collected include the annual precipitation of 6 meteorological stations covering the study area (see Tab. S.1 in Supplementary Material) and temperature data from Bni Mathar (1970–2016) and Oujda (1935–2020) stations. This study, including its data collection and processing phases, was conducted in full and strict compliance with the ethical rules and values in research, stipulated in the ethics and scientific integrity charter of the National Institute of Agricultural Research of Morocco. The collection of field data required, beforehand, the voluntary participation and the explicit consent of the participants, to whom were provided detailed information on the objective of the study, a guarantee of the confidentiality of the information collected as well as the use of statements and opinions of participants in the preparation of a scientific paper.

### 2.4 Data analysis methods

Historical climate data recorded by meteorological stations were examined using statistical methods and graphical representations to highlight any changes in precipitation and temperature patterns. The Mann-Kendall test was conducted to assess the trends in the values observed in the climate series studied for a given climate parameter. The null hypothesis to be tested is the absence of a trend in the time series considered (Sulaiman et al., 2015; Asare-Nuamah and Botchway, 2019). In order to identify possible climatic ruptures within meteorological series, two homogeneity tests were used, namely the Pettitt test (Pettitt, 1979) and the Buishand test (Buishand, 1982). The null hypothesis of these tests is the absence of rupture. These statistical methods aim to show a change of the average behavior of the studied climatic parameter (rainfall, temperature). According to Lubès et al. (1994), a rupture is defined as a change in the probability law of the chronological series at a given moment. In order to identify the years of drought within the rainfall series studied, we calculated for each station the Standardized Precipitation Index (SPI). The SPI is an index that allows the definition of drought and its monitoring (Khan et al., 2017). Positive SPI values designate rainfall amounts greater than the median (wet periods) and negative values denote drier conditions (less than the median precipitation) (Tigkas et al., 2015). The mathematical formula of SPI established by McKee et al. (1993), is as follows:

(1) SPI = (Pi – Pa) / σ

Where Pi: Precipitation of year i; Pa: average precipitation; σ: Standard deviation.

To analyze the perceptions of pastoralists with regard to CC, we have used their statements which represent their local knowledge and their lived experiences. Thus, descriptive statistics were generated in particular as regards the main perceived CC manifestations. Data on adaptation were analyzed using descriptive statistics, the chi-square test of homogeneity, Kruskal-Wallis and Mann-Whitney tests. The Chi-square homogeneity test was performed to highlight the differences in the adoption of CC adaptation strategies between three categories of pastoralists (small, medium and large). The level of significance was set at *p* ≤ 0.05. To measure the strength of relationship between these two nominal variables, the Cramer's V correlation coefficient is used. A value close to zero indicates a weak association while a high value of this coefficient reveals a strong relationship between these nominal level variables. The Mann-Whitney (Gold, 2007; Nachar, 2008) and Kruskal-Wallis (Guo et al., 2013; Ostertagova et al., 2014) tests are suitable nonparametric methods to compare two or more independent samples and, therefore, check if they come from the same distribution. The null hypothesis is that there is no significant difference between groups (pastoralists' categories) in terms of the frequency of adoption of CC adaptation practices. If the Kruskal-Wallis test is significant (*p* ≤ 0.05), the Mann-Whitney U test is run to identify categories of pastoralists that are statistically different from each other.

In order to identify the key factors influencing the adoption of the main adaptation practices to CC used by pastoralists in the high plateaus of eastern Morocco, a binary logistic regression analysis was performed. This regression method is the most appropriate and commonly used in adoption decision studies involving binary choices such as Kgosikoma et al. (2018); Hirpha et al. (2020) and Idrissou et al. (2020). According to Nabikolo et al. (2012) and Idrissou et al. (2020), the equation of the binary logit model is presented as follows:

(2) Y = *β*_0_ + *β*_1_X_1i_+ *β*_2_X_2i_ +… + *β*_16_X_16i_

Where: Y = the dichotomous dependent variable indicates whether or not a pastoralist has adopted the adaptation practice considered; i: the i^th^ observation (pastoralist); *β*_0_: the intercept term; *β*_1_- *β*_16_: the coefficients of the predictors to be estimated; X_j_: the explanatory variables (_j_: 1, ….,16). The explanatory variables used in the logistic regression models are socioeconomic attributes (age of the pastoralist, educational level, size of household, number of equipment owned, ...), institutional factors (access to credit and training, membership of a local professional organization), climate perception factors (perception of changes in rainfall, temperature and sandstorms) and geographic variables (agroecological location) (see Tab. S.2 in Supplementary Material). The effect of these predictors on the adoption of CC adaptation strategies differs according to the measure embraced. Thus, a univariate model was estimated for each choice within a set of nine endogenous coping and adaptation practices largely used by the pastoralists in the study area, namely mixed-species rearing, pastoral mobility, storage of animal feed, climate multihazard insurance, sale of livestock in good physical condition, practice of livestock fattening, regular sale of livestock to buy animal feed, converting livestock capital into real estate investment and casual wage work. The fit of the regression models obtained was checked by the chi-square test (χ^2^). The goodness of fit of the final models was assessed using the Hosmer-Lemeshow test (Archer and Lemeshow, 2006). For both tests, the significance level was 0.05.

## 3 Results and discussion

### 3.1 Characteristics of respondents

Summary statistics of the explanatory variables used in the logistic regression analysis are given in Tab. S.3 (see Supplementary Material). The average age of respondents is 52 years, with the majority (60%) aged 50 years or older. The pastoralists interviewed largely lack any formal education (71%). The average household size is 8 persons. This indicates a potential for active labor force in livestock rearing. The average number of hired laborers available (shepherds, agricultural workers) is low and does not exceed 2 persons. On average, the cultivated area is 31 hectares. Cultivated areas in the HPEM are continuously increasing, by means of land clearing and rangelands cultivation, due to increasing population pressure and the desire to individually appropriate portions of collective pastoral lands (Maâtougui et al., 2011; Bechchari et al., 2014). The average number of sheep and goats per household are about 167 and 33 heads, respectively. Just over half own at least one head of cattle. The livestock herd size provides information on the wealth status of the household and its financial capacity to adopt new technologies including those related to CC adaptation (Deressa et al., 2009; Opiyo et al., 2015). More than 61% of pastoralists have at least one piece of agricultural equipment or mean of transport such as trucks, tractors and water tanks.

The proportion of pastoralists able to access formal credit does not exceed 23%. On the contrary, they obtain informal credit from speculators or resellers of livestock feed, thus enabling them to acquire the feed necessary for the nutrition of their herds. This is due to problems of creditworthiness, but especially to a mismatch of the financial facilities offered by the banks, which do not make finance available for mobile or transhumant livestock rearing. Arimi (2014) highlighted that the poor access of farmers to conventional credit services, could discourage them from implementing adapted and improved techniques in the face of CC. The proportion of pastoralists who have received training from external bodies, mainly on livestock rearing and rangeland management is low, i.e., 20%. Only 26% are members of the formal livestock producers' organization, as the National Association of Sheep and Goat Herders (ANOC). Regarding climate perceptions, pastoralists have noticed several variations in their recent climatic conditions compared to the period before 1970, namely an important reduction in annual rainfall totals and increase in temperature and extreme weather events (i.e., droughts, heavy rains, high winds and sandstorms). This indicates that the majority of respondents clearly perceived major changes that have occurred in recent decades in their usual climatic conditions and therefore, they may be more likely to adapt. In line with this finding, previous studies revealed that farmers’ perceptions toward CC influence the implementation and success of adaptation measures (Deressa et al., 2009; Gbetibouo, 2009; Tesfahunegn et al., 2016).

### 3.2 Analysis of data recorded at meteorological stations

#### 3.2.1 Variability, trends and ruptures in rainfall

Precipitation in the study area is characterized by high variability both temporally and spatially. Rainfall fluctuates from one year to another with high variation coefficients ranging from 28% to 52% (Table 3). The coefficient of variation increases and annual rainfall diminishes the further south one goes. Indeed, the average annual rainfall varies from 275 mm for Oujda (semi-arid zone) to 120 mm for Figuig (arid or pre-Saharan zone).

**Table 3:** Summary of precipitation for the 6 meteorological stations (1981–2019).

Stations	Min (mm)	Max (mm)	Mean (mm)	SD (mm)	CV (%)
Oujda	151.3	465.7	274.6	75.7	27.6
Taourirt	64.6	436.7	191.0	68.7	36.0
Bni Mathar	76.6	281.7	184.8	56.1	30.4
Tendrara	49.8	391.7	196.0	101.9	52.0
Bouaârfa	57.1	277.3	146.2	62.3	42.6
Figuig	27.0	328.0	119.3	61.1	51.2

Note: SD: Standard deviation, CV: Coefficient of variation.

Rainfall series considered clearly indicated a general downward trend in the annual amounts of precipitation (Table 4). In order to find out if there is a significant trend within the rainfall series studied, a Mann- Kendall test was carried out. The precipitation series of Bni Mathar, Taourirt and Oujda stations, show a decreasing tendency, statistically significant, unlike rainfall series from other meteorological stations.

**Table 4:** Mann-Kendall trend test results for annual precipitations.

Stations	Kendall's Tau-B	Sig.	Number of years	Trend
Oujda	-0.189∗∗	0.004	106	Decreasing
Taourirt	-0.217∗∗	0.002	97	Decreasing
Bni Mathar	-0.194∗∗	0.007	89	Decreasing
Tendrara	-0.075	0.303	87	Decreasing
Bouaârfa	-0.001	0.990	39	Decreasing
Figuig	-0.057	0.441	85	Decreasing

Note: ∗∗: The correlation is significant at the 0.01 level (two-tailed).

**Figure 2:**
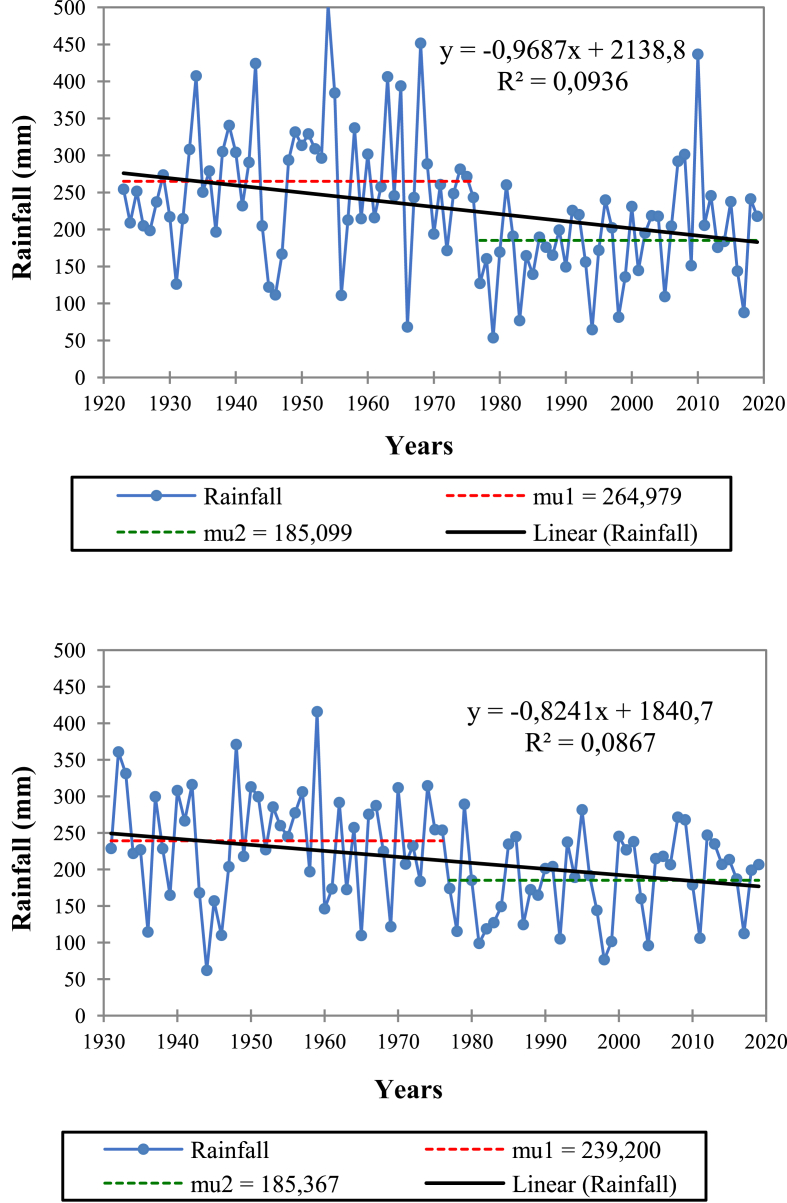
Homogeneity of annual rainfall according to the Pettitt's test at the Taourirt (top) and Bni Mathar (bottom) stations.

The results obtained by Pettitt's test (K = 853.000, *p* = 0.002; K = 1322.000, *p* < 0.0001; K = 1290.000, *p* = 0.000) and Buishand's test (Q = 16.639, *p* = 0.002; Q = 21.572, *p* < 0.0001; Q = 20.237, *p* = 0.000), respectively for the stations of Bni Mathar, Taourirt and Oujda, showed that their rainfall series were not homogenous. Therefore, there is a point or a time t from which there is a significant change in the mean of annual rainfall, this point is at 1976 for Bni Mathar and Taourirt (Figure 2) and 1980 for Oujda.

Overall, a general regression trend of the annual rainfall amounts is observed between the two recording periods before and after the 1976 rupture date (Table 5). In fact, the mean annual rainfall has decreased, on average, by more than 18% or 46 mm. The northern part of the study area was the most affected by this decrease in annual precipitation over the periods considered, since the stations of Taourirt, Oujda and Bni Mathar recorded the largest decreases with respectively 80 mm, 77 mm and 54 mm.

**Table 5:** Means' evolution of annual precipitation before and after rainfall rupture (1976).

Stations	Average before rupture (mm)	Average after rupture (mm)	Difference (mm)	Difference (%)
Oujda	355.4	278.0	-77.4	-21.8
Taourirt	264.0	184.0	-80.0	-30.0
Bni Mathar	239.0	185.0	-54.0	-23.0
Tendrara	202.0	196.0	-7.0	-3.0
Figuig	133.2	119.8	-13.4	-10.1

The findings related to the drop in annual rainfall since the mid-1970s are supported by earlier studies (Jorion, 2009; Mahyou et al., 2010; Maâtougui et al., 2011; Bechchari et al., 2014; François et al., 2016; Melhaoui et al., 2018; Snaibi, 2020b). After noting a general downward trend in precipitation from the mid-1970s, Jorion (2009) indicated that the significant decline in annual rainfall concerned stations in the northern part of the study area, namely Taourirt (1922–2007), Oujda (1917–2008) and Bni Mathar (1932–2006) with respectively a decrease in annual precipitation of 38.5% (- 105 mm), 24.8% (- 88 mm) and 23% (−55 mm) and dates of rupture located in 1976, 1976 and 1980. Similarly, Melhaoui et al. (2018), on the basis of the rainfall series relating to the stations of Bni Mathar (1935–2015), Tendrara (1935–2015), Bouaârfa (1980–2015) and Figuig (1935–2015), did not identify any rupture of rains, except the one detected at the Bni Mathar station, which is located at 1976.

#### 3.2.2 Occurrence of drought in the study area

The results of the calculations of the SPI from the annual rainfall data recorded by the meteorological stations studied, indicated that the frequency of droughts is quite high with an average of 36%, or more than one year of drought every three years. This frequency of dry years has increased significantly in recent decades, showing an average increase from 26% to 49% between the two periods before and after the 1976 rupture date (Table 6). From 1976, the frequency of droughts fluctuated between 40% (Tendrara) and 62% (Oujda). The northern part of the study area was the most affected, since the stations of Oujda, Taourirt and Bni Mathar exhibited the largest increases in the frequency of drought, i.e. 182% (from 22% to 62%), 136% (from 22% to 52%) and 105% (from 21% to 43%) respectively. These findings are in agreement with those previously presented which showed a downward trend in annual rainfall amounts over the past five decades. Thus, there is a persistence of the occurrence of rainfall deficits which can be interpreted as the accentuation and persistence of the drought that became prominent from the mid-1970s.

**Table 6:** Statistics of drought, normal and wet years according to the SPI.

Periods	Before 1976			After 1976		
Stations	Drought	Normal	Wet	Drought	Normal	Wet
Oujda	22% (14)	30% (19)	48% (30)	62% (26)	24% (10)	14% (6)
Taourirt	22% (12)	22% (12)	56% (31)	52% (22)	31% (13)	17% (7)
Bni Mathar	21% (10)	20% (9)	59% (27)	43% (18)	36% (15)	21% (9)
Tendrara	35% (16)	18% (8)	47% (21)	40% (16)	30% (12)	30% (12)
Figuig	31% (13)	21% (9)	48% (20)	45% (19)	26% (11)	29% (12)
Average (in %)	26	22	52	49	29	22

Note: The number in brackets indicates the number of years.

This obtained result is supported by several previous studies which have highlighted increased frequency of droughts in the study area in recent decades (Jorion, 2009; Mahyou et al., 2010; Maâtougui et al., 2011; Bechchari et al., 2014; Melhaoui et al., 2018). Jorion (2009), using Lamb's rainfall anomaly index, found that the values of this index since the late 1970s were negative, indicating recurring precipitation deficits and more frequent droughts in the study area. Melhaoui et al. (2018), based on the calculation of the standardized precipitation index relating to rainfall series 1980–2015 from the stations of Bni Mathar, Tendrara, Bouaârfa and Figuig, underlined that the frequency of dry years for the whole of these stations oscillated between 33% and 52%.

#### 3.2.3 Temperature trends and ruptures

**Figure 3:**
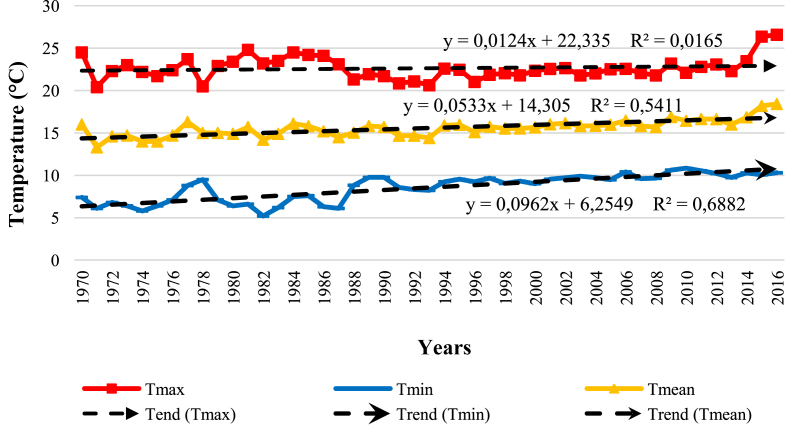
Trend of the mean, minimum and maximum annual temperature at the Bni Mathar station.

According to the Mann-Kendall test, annual mean temperatures series of Bni Mathar (1970–2016) and Oujda (1935–2020) stations showed a significant upward trend (τb = 0.553, *p* < 0.0001; τb = 0.311, *p* < 0.0001). Likewise, the minimum temperature series exhibited a significant increasing tendency for the stations of Bni Mathar (τb = 0.647, *p* < 0.0001) and Oujda (τb = 0.333, *p* < 0.0001). Besides, the series of annual mean temperatures showed a significant change in the mean observed, respectively, for the stations of Bni Mathar and Oujda, according to Pettitt's test (K = 424.000, *p* < 0.0001; K = 1343.000, *p* < 0.0001) and Buishand's test (Q = 15.047, *p* < 0.0001; Q = 27.252, *p* < 0.0001), with rupture dates located at 2000 and 1993. Thus, the mean temperature increased from 15.12 to 16.49 °C in Bni Mathar and from 16.841 to 18.126 °C in the station of Oujda, for the periods before and after the respective rupture dates considered. The null hypothesis of homogeneity of the minimum temperature data was rejected for the stations of Bni Mathar and Oujda, according to the tests of Pettitt (K = 502.000, *p* < 0.0001; K = 1298.000, *p* < 0.0001) and of Buishand (Q = 19.608, *p* < 0.0001; Q = 25.460, *p* < 0.0001). In fact, the minimum temperature recorded the greatest increase either from 6.954 to 9.655 °C between the period before and after the rupture date of 1988 for Bni Mathar, and from 10.046 to 11.150 °C, with a rupture date located at 1986 for the station of Oujda. Figure 3 clearly indicated this increasing temperature trend over the last decades, mainly of the minimum temperature since its modification factor was the highest, i.e., 0.096 compared to those of the mean (0.053) and maximum (0.012) temperatures. This observation of the increase in temperature is corroborated by previous studies (e.g., Jorion, 2009; François et al., 2016; Snaibi, 2020b).

### 3.3 Comparison between pastoralists' perceptions of CC and actual observed long-term changes in climate

**Figure 4:**
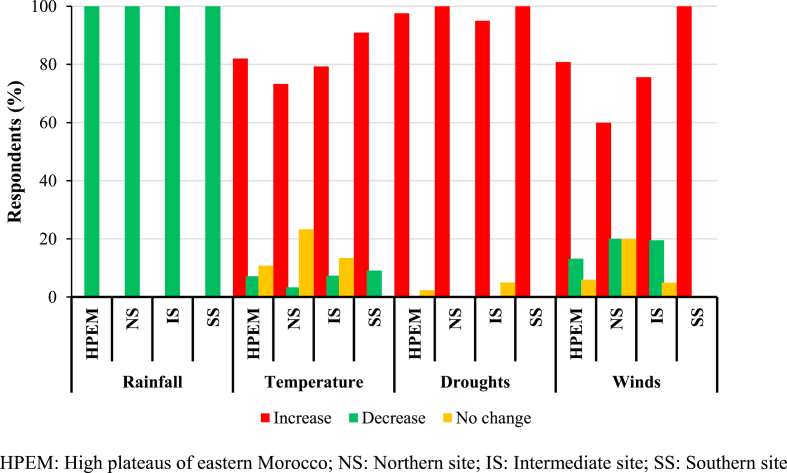
Pastoralists' perceptions of the main changes in climatic conditions that have occurred in recent decades (n = 167).

The results relating to the perceptions of pastoralists regarding climate variability and change are presented in Figure 4. The pastoralists surveyed were asked whether they have perceived any changes in their current climate in comparison with that of the last five decades. All of the pastoralists interviewed have observed a significant drop in the total annual rainfall, particularly from the 1970s. In fact, this important regression in the amounts of rainfall was added to the great interannual variability of precipitation (high values of the coefficient of variation ranging from 34% to 47%), which currently characterizes the climate of the high plateaus of eastern Morocco. The result is an increased vulnerability of the already very fragile vegetation cover, which does not allow stability of fodder production of the steppe pastures from one year to the next. The main consequences of the decline in precipitation, from the perspective of pastoralists, relate to the reduction in the fodder production of rangelands and the yields of cereal crops, the decrease in water resources available for watering of livestock and also disruption of the usual schedule of animal husbandry and agricultural practices in general. In addition, the majority of respondents (more than 97%) noted an important disruption of the rainy season, which resulted in the reduction of rainy days, a delayed onset of the rainy season and the appearance of frequent dry spells during this one. Indeed, according to Jorion (2009) and Melhaoui et al. (2018), the decline in total annual rainfall is due to a considerable drop in precipitation in the spring season and to a lesser extent in the winter season, hence seasonal droughts which became more frequent during these two seasons. This change in seasonal rainfall clearly would result in a reduction in the length of the growth period of pastoral plants and cereal crops, since vegetative development will therefore be limited and cannot extend later in the year (beyond the month of May). In addition, plant propagation and seed production will therefore be negatively affected or even slowed down. Furthermore, a significant proportion of the surveyed pastoralists (67%) perceived an increase in heavy rains leading to flooding, with considerable damage on the herds, tents and road infrastructure.

Over 91% of respondents noted a significant change in the temperature regime during the last five decades. In fact, the majority of pastoralists (82%) observed an increase in temperature, particularly in the southern area (91%). Decreased rainfall combined with high summer temperatures has resulted in a desiccation of the local climate with negative repercussions on plant cover and the availability of water resources. In addition, the increase in the occurrence of droughts was noted by a large majority of interviewees, i.e. 97%. Therefore, these statements by pastoralists confirm the results obtained above and those in the secondary literature (Born et al., 2008; Jorion, 2009; Maâtougui et al., 2011; François et al., 2016; Melhaoui et al., 2018; Snaibi, 2020b). Likewise, these local perceptions were found to be similar to those reported in earlier studies which indicated a decreasing trend in rainfall and an increase in temperature over the last decades in many African countries (Gbetibouo, 2009; Ouédraogo et al., 2010; Vissoh et al., 2012; Tamiru et al., 2014; Opiyo et al., 2015; Ndamani and Watanabe, 2016).

The increase in the frequency of strong winds, sometimes causing damaging sandstorms, was cited by a large proportion of respondents (81%), particularly those in the southern area. This statement is supported by Mahyou et al. (2016) and Melhaoui et al. (2018) who indicated that high, hot and dry winds have become more and more frequent during the summer, especially in the southern part of the study area, giving rise to significant sandstorms. Similarly, an increase in the occurrence of high winds, mainly during the dry season, has been observed in the south of Benin (Baudoin, 2010; Vissoh et al., 2012) and the northeast of Nigeria (Yila and Resurreccion, 2013). Hence, we can conclude that pastoralists’ perceptions are perfectly consistent with the climatic trends actually observed in the high plateaus of eastern Morocco.

### 3.4 Adaptation practices to CC according to socio-economic categories

The ten adaptation practices in response to climate variability and change used in the analysis which follows, constitute those that are most widespread in the high plateaus of eastern Morocco in accordance with the statements of pastoralists surveyed and reported in some previous studies (Bechchari et al., 2014; Snaibi, 2020a). The frequencies of these main adaptation and coping practices differed among categories of pastoralists (Table 7). Indeed, large-scale pastoralists, in comparison with other categories, show high levels of adoption of the great majority of adaptation practices. They have opted for strategies aimed at diversifying their production (combination of animal husbandry and grain crops, raising of sheep and goats in mixed herds), adjusting livestock management (storage of animal feed, increased mobility to favorable grazing sites, fattening practice), orienting livestock production towards the market (animals with good physical conformation and better taste qualities), investing in real estate to circumvent climatic and economic insecurity, and taking out insurance against multiple climatic risks. However, all these adaptation actions involve significant financial resources, which explains their low adoption rate, especially among small-scale pastoralists. On the contrary, as might be expected, it is the small-scale pastoralists who frequently resort to occasional wage labor in addition to animal husbandry, as a traditional coping practice, to meet their needs and those of their herds.

**Table 7:** Results of the Chi-square test of homogeneity.

Adaptation practices	Pastoralist categories			χ^2^ Pearson (Significance)	Cramer's V
	Small (n = 96)	Medium (n = 47)	Large (n = 24)		
Livestock-grain crop mixed system∗∗				14.384 (0.001)	0.293 (M)
Yes	71	44	24		
No	25	3	0		
Mixed-species rearing (sheep & goats)				4.718 (0.095)	0.168 (L)
Yes	69	40	21		
No	27	7	3		
Climate insurance∗∗∗				36.493 (0.000)	0.467 (S)
Yes	27	33	20		
No	69	14	4		
Livestock feed storage∗∗				11.865 (0.003)	0.267 (M)
Yes	35	30	15		
No	61	17	9		
Frequent sale of livestock to buy animal feed				0.917 (0.632)	0.074 (L)
Yes	43	25	11		
No	53	22	13		
Sale of livestock in good physical condition∗∗				14.273 (0.001)	0.292 (M)
Yes	32	23	18		
No	64	24	6		
Pastoral mobility∗∗∗				20.466 (0.000)	0.350 (M)
Yes	26	23	18		
No	70	24	6		
Conversion of livestock into real estate investments∗∗∗				17.355 (0.000)	0.322 (M)
Yes	28	21	18		
No	68	26	6		
Casual salaried work∗∗				13.736 (0.001)	0.287 (M)
Yes	41	13	1		
No	55	34	23		
Livestock fattening practice∗∗∗					
Yes	20	15	16	19.072 (0.000)	0.338 (M)
No	76	32	8		

Note: Significance: ∗∗∗P < 0.001, ∗∗P < 0.01; ∗P < 0.05. Degree of freedom = 2. Cramer's V (Strength of relationship): S: Strong (between 0.40 and 0.80); M: Moderate (between 0.20 and 0.40); L: Low (between 0.10 and 0.20).

To find out whether there are significant differences between the three categories of pastoralists (small, medium and large) in terms of adoption frequency of each CC adaptation practice, the chi-square test of homogeneity was used. The null hypothesis of this non-parametric test indicates that the three groups of pastoralists are homogeneous with respect to the proportions of the modalities of the dichotomous dependent variable that is the adaptation measure considered (adopted or not adopted). If the null hypothesis is rejected, it can be argued that these proportions are different in the observed groups of pastoralists. The Table 7 revealed that the distribution of the overwhelming majority of the adaptive practices implemented differed significantly from one category of pastoralists to another. The strength of the relationship between types of adaptation practices and categories of pastoralists is, in most cases, moderate with Cramer's V values ranging from 0.267 to 0.350.

The Kruskal-Wallis test showed that the adoption' frequency of CC adaptive practices differed significantly between the categories of pastoralists (χ^2^(2) = 7.603, *p* = 0.022), with a mean rank of 9.80 for small-scale pastoralists, 16.10 for medium pastoralists and 20.60 for large-scale pastoralists. This large difference in mean ranks between these three groups indicated that large-scale pastoralists are more likely to adopt adaptation measures. In addition, the results of the Mann-Whitney U test showed that the differences in adopting CC adaptation practices are significant between small-scale and large-scale pastoralists (U = 16.000, *p* = 0.009). The effect size calculated by dividing the absolute standardized test statistic Z which equals 2.574 by the square root of the number of pairs (N = 20) was 0.575. This is therefore a large effect according to Cohen's classification (1988) of effect sizes which states small effect size (0.10–0.30), moderate effect (0.30–0.50) and large effect for a measurement greater than or equal to 0.50. However, the differences between small-scale and medium pastoralists on the one hand (U = 27.000, *p* = 0.089) and between medium and large-scale pastoralists on the other hand (U = 33.000, *p* = 0.218) turned out to be insignificant. Since the differences in frequency adoption of CC adaptation practices between small-scale and large-scale pastoralists are significant and the distribution of most of these practices also differed significantly between these two groups, therefore, the study suggests that pastoralists' adaptation depends on their respective wealth status expressed in term of the size of the sheep flock held. This finding is supported by previous studies that have indicated that the adaptive behaviour of livestock producers is strongly influenced by the size of the herd owned (Bechchari et al., 2014) and that a large herd size is a determining factor affecting the adaptation of pastoralists in response to CC (Balew et al., 2014; Zampaligre' et al., 2014; Berhanu and Beyene, 2015; Opiyo et al., 2015).

### 3.5 Factors influencing the implementation (adoption) of CC adaptation measures

The results from the binary logistic regression analysis of the survey data are presented in Table 8. Given that the overall correct percentage prediction rate has high values, ranging from 74% to 87%, this means that the regression models gained have high quality and strength thus enabling to correctly and successfully classify the observations (pastoralists). The classification accuracy is significantly better than that obtained by chance, and the predictors therefore jointly contribute to explaining a significant part of the variability in the outcome variable, which is the adopted adaptation measure. In addition, the most important factors, which significantly affect CC adaptation practices at the household level, are equipment, educational level of household head, size of livestock herd, household size, training received, perception of climate change and agroecological location.

**Table 8:** Binary logistic regression results on determinants of pastoralists’ adaptation to CC in the HPEM.

Predictors	β	SE β	Wald's χ2	df	p	Odds ratio (exp β)
**Mixed-species rearing**						

Constant	0.858	1.343	0.408	1	0.523	2.358
Age	-0.035	0.020	3.015	1	0.082	0.965
Educational level	1.253	0.624	4.034	1	0.045	3.500
Household size	0.164	0.096	2.921	1	0.087	1.178
Equipment	0.362	0.206	3.102	1	0.078	1.436
Training	-2.168	0.762	8.097	1	0.004	0.114
Northern site	-1.977	0.802	6.073	1	0.014	0.138
Intermediate site	1.587	0.867	3.347	1	0.067	4.889
*Test*						
*Overall model evaluation*			45.457	16	0.000	
*Goodness-of-fit*^*1*^			10.839	8	0.211	
*Correct percentage of prediction*: 84.4%						

**Pastoral mobility**						

Constant	-2.562	1.203	4.536	1	0.033	0.077
Sheep herd size	0.004	0.002	3.896	1	0.048	1.004
Equipment	0.325	0.178	3.345	1	0.067	1.385
Access to formal credit	-0.937	0.541	2.998	1	0.083	0.392
Training	1.402	0.582	5.800	1	0.016	4.062
Heavy rains	2.211	0.658	11.297	1	0.001	9.127
Intermediate site	-1.214	0.667	3.306	1	0.069	0.297
*Test*						
*Overall model evaluation*			68.438	16	0.000	
*Goodness-of-fit*^*1*^			5.281	8	0.727	
*Correct percentage of prediction*: 76.6%						

**Livestock feed storage**						

Constant	-2.698	1.393	3.753	1	0.053	0.067
Household size	0.145	0.084	2.949	1	0.086	1.156
Access to formal credit	2.212	0.615	12.947	1	0.000	9.133
Training	-1.159	0.668	3.009	1	0.083	0.314
Membership in LPO	-1.113	0.593	3.521	1	0.061	0.329
Heavy rains	1.890	0.828	5.214	1	0.022	6.621
Northern site	3.495	0.970	12.977	1	0.000	32.957
Intermediate site	-1.451	0.781	3.456	1	0.063	0.234
*Test*						
*Overall model evaluation*			99.242	16	0.000	
*Goodness-of-fit*^*1*^			6.463	8	0.596	
*Correct percentage of prediction*: 84.4%						

**Climate insurance**						

Constant	-3.378	1.366	6.119	1	0.013	0.034
Labor force	-0.680	0.225	9.174	1	0.002	0.506
Farm size	0.018	0.010	3.304	1	0.069	1.018
Sheep herd size	0.009	0.003	11.505	1	0.001	1.009
Temperature change	-1.230	0.722	2.899	1	0.089	0.292
Heavy rains	1.894	0.679	7.784	1	0.005	6.648
Sandstorms	1.391	0.809	2.960	1	0.085	4.020
Intermediate site	-2.209	0.733	9.076	1	0.003	0.110
*Test*						
*Overall model evaluation*			82.283	16	0.000	
*Goodness-of-fit*^*1*^			20.791	8	0.008	
*Correct percentage of prediction*: 78.4%						

**Sale of livestock in good physical condition**						

Constant	-1.920	1.174	2.674	1	0.102	0.147
Size of cattle flock	0.113	0.064	3.145	1	0.076	1.120
Northern site	2.312	0.752	9.449	1	0.002	10.096
Intermediate site	2.279	0.727	9.833	1	0.002	9.764
*Test*						
*Overall model evaluation*			59.818	16	0.000	
*Goodness-of-fit*^*1*^			14.539	8	0.069	
*Correct percentage of prediction*: 74.3%						

**Livestock fattening practice**						

Constant	-0.413	1.221	0.114	1	0.735	0.662
Educational level	1.162	0.528	4.839	1	0.028	3.198
Equipment	0.735	0.222	10.968	1	0.001	2.085
Training	1.621	0.590	7.534	1	0.006	5.056
*Test*						
*Overall model evaluation*			69.462	16	0.000	
*Goodness-of-fit*^*1*^			2.518	8	0.961	
*Correct percentage of prediction*: 83.2%						

**Sale of livestock to buy animal feed**						

Constant	-0.828	0.971	0.727	1	0.394	0.437
Labor force	-0.437	0.182	5.774	1	0.016	0.646
Access to formal credit	-1.076	0.476	5.108	1	0.024	0.341
Northern site	2.338	0.701	11.116	1	0.001	10.361
*Test*						
*Overall model evaluation*			39.643	16	0.001	
*Goodness-of-fit*^*1*^			2.805	8	0.946	
*Correct percentage of prediction*: 70.7%						

**Investments in real estate**						

Constant	-3.978	1.723	5.332	1	0.021	0.019
Age	0.065	0.024	7.000	1	0.008	1.067
Educational level	1.232	0.649	3.610	1	0.057	3.430
Size of cattle flock	0.175	0.094	3.492	1	0.062	1.191
Equipment	0.688	0.224	9.434	1	0.002	1.989
Membership in LPO	-2.356	0.766	9.464	1	0.002	0.095
Temperature change	-2.215	0.954	5.387	1	0.020	0.109
Heavy rains	4.423	0.971	20.745	1	0.000	83.324
Intermediate site	-5.472	1.067	26.316	1	0.000	0.004
*Test*						
*Overall model evaluation*			119.184	16	0.000	
*Goodness-of-fit*^*1*^			4.502	8	0.809	
*Correct percentage of prediction*: 86.8%						

**Casual salaried work**						

Constant	2.479	1.330	3.476	1	0.062	11.929
Educational level	-1.212	0.614	3.897	1	0.048	0.298
Labor force	0.500	0.303	2.728	1	0.099	1.649
Equipment	-1.070	0.322	11.005	1	0.001	0.343
Access to formal credit	1.284	0.604	4.516	1	0.034	3.610
Temperature change	-1.489	0.870	2.929	1	0.087	0.225
Sandstorms	1.639	0.876	3.501	1	0.061	5.152
Northern site	-3.770	0.978	14.874	1	0.000	0.023
*Test*						
*Overall model evaluation*			86.181	16	0.000	
*Goodness-of-fit*^*1*^			23.288	8	0.003	
*Correct percentage of prediction*: 83.2%						

Notes: *β*: Estimated coefficient; Exp (*β*): Exponential beta gives the odd ratio of the outcome variable; LPO: Livestock producers' organization; ^1^: Hosmer and Lemeshow goodness-of-fit test; Number of observations: 167.

#### 3.5.1 Possession of equipment

The results indicate that the pastoralists’ endowment with agricultural and transport equipment (trucks, tractors, water tanks and motor pump) significantly facilitates their adoption of several adaptation practices in the face of CC. Indeed, for a one standard deviation positive change in total number of equipment in ownership, holding other explanatory variables constant, the results reveal an increase in the implementation of mixed-species rearing, pastoral mobility, livestock fattening practice and investments in real estate as CC adaptation measures by 0.362, 0.325, 0.735 and 0.688 standard deviations respectively. Thus, the study suggests that the more agricultural and transport equipment a pastoralist has at his disposal, the more likely he is to adapt to the negative effects of CC. This finding is consistent with other previous studies, which have shown that the ownership of heavy machinery or agricultural equipment in general, plays a positive and a significant role in the rural farming adaptation toward climate change (Hassan and Nhemachena, 2008; Ouédraogo et al., 2010; Zampaligre' et al., 2014).

#### 3.5.2 Educational level

The level of education reached by the pastoralist positively and significantly influences the likelihood that he will undertake some CC adaptation responses, namely the diversification of animal species, the practice of fattening livestock and the conversion of livestock capital into land capital. Other studies corroborate this finding by showing that the education level is a key factor affecting the farmers’ adaptation in response to CC (Opiyo et al., 2015; Ndamani and Watanabe, 2016; Asfaw et al., 2019; Idrissou et al., 2020). In contrast, this factor has been found to be a non-significant determinant of CC adaptation, according to Arimi (2014), Balew et al. (2014), Berhanu and Beyene (2015).

#### 3.5.3 Sheep herd size

Regression analysis results indicate that a positive change of one standard deviation in the size of sheep herd in ownership, holding the other explanatory variables constant, increases the uptake of climate insurance and pastoral mobility as CC adaptation practices by 0.009 and 0.004 standard deviations respectively. Therefore, the size of the sheep herd positively and significantly affects the likelihood that pastoralists will adopt the two relevant adaptation measures aforementioned. The study shows that this factor plays a determining role in the diversification of adaptation portfolios among pastoralists. In addition, the smaller the size of the sheep herd, the more the pastoralist chooses occasional wage labor as an alternative livelihood in response to perceived climatic changing. In line, many previous studies have pointed out that livestock herd size has a positive and significant impact on the probability of farmers' adopting CC adaptation strategies (Bechchari et al., 2014; Zampaligre' et al., 2014; Opiyo et al., 2015; Idrissou et al., 2020).

#### 3.5.4 Household size

Given that the household size factor coefficients for mixed-species rearing and livestock feed storage are positive, this means that households with a large number of members are more likely to use these two adaptation techniques than those with small numbers. Indeed, a large number of household members could provide information on the availability of family labor, which could be used in the implementation of these labor-intensive adaptation practices. Previous studies have shown that family size positively affects farmers' ability to undertake CC adaptation strategies (Ndamani and Watanabe, 2016; Opiyo et al., 2015; Atinkut and Mebrat, 2016; Taruvinga et al., 2016). In contrast, Deressa et al. (2009) and Nabikolo et al. (2012) found that household size is not an important factor influencing farmers’ choice and decision in adapting to CC in the Nile Basin of Ethiopia and Eastern Uganda respectively.

#### 3.5.5 Training

The logistic regression results reveal that trained pastoralists are more likely to embrace two relevant adaptation techniques, namely livestock fattening practice and pastoral mobility. Meanwhile, this factor had a negative and significant impact on the likelihood to adopt the raising of mixed flocks of sheep and goats, a traditional and widespread adaptation practice. Previous studies highlighted that the training received by the head of the household increases the adoption of improved CC adaptation strategies (Piya et al., 2013; Tiwari et al., 2014; Asfaw et al., 2019). On the contrary, Yila and Resurreccion (2013) underlined that this factor is not a significant determinant influencing the farmers’ decision to adapt to CC.

#### 3.5.6 Perception of long-term change in climatic conditions

The results indicate that local perception relating to CC significantly influenced adaptation in the face of this climate phenomenon. This finding is in line with those reported by other previous studies (Debalke, 2011; Balew et al., 2014; Ndamani and Watanabe, 2016). Indeed, the pastoralists’ perception of changes in climatic parameters (rainfall, temperature and sandstorms) was found to have a significant role in adopting climate insurance, pastoral mobility and livestock feed storage as CC adaptation measures. This indicates that these adaptation practices are specific responses to CC.

With reference to the regression models, the obtained results show that the perception of heavy rains significantly increases the likelihood of implementing the adaptive practices of pastoral mobility, climate insurance, livestock feed storage and conversion of livestock capital into land capital. Therefore, the pastoralists' who perceived a change in rainfall regime over the last decades are more likely to cope with the CC effects. Berhanu and Beyene (2015) expressed that the perception towards changes in rainfall pattern influences positively and significantly the pastoral household's decision to embrace the herd mobility as a key adaptation practice. Likewise, Balew et al. (2014) pointed out that the farmers' perception on rainfall changes (floods) affects their decisions about which CC adaptation practices they implement among a range of existing options. Furthermore, many preceding studies revealed that farmers' perceptions regarding long-term changes in rainfall influence their decisions in response to CC and their choices of adaptation actions to be undertaken (Hassan and Nhemachena, 2008; Debalke, 2011; Piya et al., 2013; Mabe et al., 2014).

The coefficient of temperature’ change perceived is negative in most cases. The results indicated that pastoralists who perceive change in temperature have a lower probability of adopting the multi-risk climate insurance, investments in real estate and casual salaried work as adaptation measures. For the other adaptive strategies, the variable "perception of temperature change" has no statistically significant effect. In the literature, the perception of long-term change in temperature is considered to be a factor having a contradictory effect on adaptation to CC. Debalke (2011) has found a negative impact of this factor on the likelihood of adoption of some adaptation practices such as soil conservation, irrigation and changing planting dates in the North Shoa Zone of Amhara Region, Ethiopia. On the contrary, other studies have pointed out that the "perceived temperature change" factor positively and significantly influenced farmers' adaptation in response to CC (Deressa et al., 2009; Mabe et al., 2014; Atinkut and Mebrat, 2016), or has been considered a non-significant determinant of adaptation (Yila and Resurreccion, 2013; Tiwari et al., 2014; Ndamani and Watanabe, 2016). However, from the perspective of pastoralists in the study area, whose main economic activity is the rearing of small ruminants on natural arid rangelands, rainfall is the key climatic factor to be considered, since the rangeland fodder production and the watering of herds depend heavily on it. In line, Mahyou et al. (2018) stated that rainfall is the major parameter influencing the pasture production in Morocco's pastoral ecosystems. In addition, the results show that the perception of increased sandstorms over the last decades positively and significantly affects the likelihood that pastoralists will sign up for climate insurance as an adaptation practice or engage in wage labor as a coping response to reduce the negative impacts of climate variability and change on their incomes. As highlighted by Balew et al. (2014), the nature of the climate factor change conditions farmers' choice of CC adaptation practices to put in place among a diverse range of available adaptation options.

#### 3.5.7 Agroecological setting

Findings indicated that living in the northern agroecological site has a significant and positive impact on the likelihood of adopting livestock feed storage, market-oriented livestock rearing and sale of livestock to purchase animal feed as adaptive practices to CC. Indeed, the northern area being endowed with abundant groundwater, this has allowed the practice of irrigated agriculture, mainly fodder crops (barley, oats, alfalfa), on a perimeter of 2,500 ha. Consequently, local pastoralists regularly store these fodder resources of agricultural origin to be used to feed the herds, particularly in times of forage shortages in natural rangelands. In addition, due to its geographical proximity to the capital of the Eastern Region of the country and above all the existence of an important local livestock market, the livestock rearing system at this site is oriented towards the market. Indeed, this locality plays the role of relay between the livestock production basins of the study area and the large animal market in the northeast region. Furthermore, living in the northern area, which exhibits the high agroecological potential, has a significant, but negative impact on the likelihood of using casual salaried work as an adaptive option, compared with the southern zone (reference site), which has limited agricultural potential and a high poverty rate.

Meanwhile, there is a higher likelihood of adopting mixed-species rearing and market-oriented livestock raising as CC adaptation practices when pastoralists live in the intermediate agroecological site. Given the vastness of the existing pastoral lands covering an area of approximately 17,360 km^2^ and their specialisation in rangeland-based small ruminants rearing, the pastoralists belonging to the intermediate agroecological site (rural territorial collectivities of Tendrara and Maâtarka) have opted to diversify their herds by rearing goats, in addition to the sheep species, and this for their characteristics of hardiness, polyfunctionality and growing urban demand for their meat. Furthermore, the local market of Tendrara constitutes a source of supply of animals for slaughter and rearing for livestock traders wishing to serve large urban areas of consumption with red meat of better quality. In addition, the conversion of livestock capital into real estate investments is more frequent in the southern area compared to the other two sites due to the low local agroecological potential combined with the conditions of environmental insecurity (increase in recurrent and prolonged droughts) and economic (instability of livestock and feed prices).

The study found that pastoralists adapt differently to CC, depending on the agroecological context in which they live. The differences observed between the three selected study sites regarding the type of CC adaptation measures implemented there, are principally due to contrasting biophysical and socio-economic conditions. Thus, the CC adaptation responses were found location specific. This finding corroborates those of many previous studies (Below et al., 2012; Bryan et al., 2013; Legesse et al., 2013; Piya et al., 2013; Tiwari et al., 2014; Zampaligre' et al., 2014; Atinkut and Mebrat, 2016; Idrissou et al., 2020).

Public CC adaptation policy aimed at improving the adaptive capacity of pastoralists in arid rangelands should provide them with affordable agricultural and transport equipment, strengthen their education and training to improve their knowledge and skills in adapting to CC, and provide them with improved and updated climate information. Since small-scale pastoralists are the most vulnerable group and the hardest hit by the negative impacts of CC, government support should therefore target them as a priority, while offering them appropriate education and vocational training that allow them to secure more skilled and regular employments.

## 4 Conclusions

The core objectives of this study were to assess whether pastoralists' perceptions of CC were consistent with climate data recorded at meteorological stations, to analyze their adaptation responses according to their socioeconomic categories and to ascertain the main factors affecting the adoption of CC adaptation measures. The statistical analysis of historical climatic data revealed a noticeable trend of decreasing precipitation and an increase in temperature and the occurrence of droughts. Pastoralists' perceptions of CC, based on their local knowledge and experiential learning, have been found to be in perfect agreement with the climate trends actually observed since the late 1970s. Indeed, all the pastoralists surveyed noted, regardless of their age, social status, agroecological site or any other influencing factors, a considerable drop in annual amounts of rainfall over the past five decades. In addition, a large majority of respondents mentioned an increase in temperature and in the frequency of droughts and high winds. These unfavorable climatic trends have led to an increase in the sensitivity and therefore the vulnerability of rangeland-based small ruminant rearing in the face of CC.

The study highlighted that among pastoralists in the high plateaus of eastern Morocco most CC adaptation practices have been adopted differentially by different socio-economic categories. Large-scale pastoralists have implemented diverse adaptation portfolios, unlike small-scale pastoralists who adopted the available adaptation actions at a low frequency and exhibited a much narrower range of options to reduce the adverse effects of CC with frequent recourse to casual wage labor. In addition, the most important factors influencing the adoption (implementation) of CC adaptation practices by pastoralists are socio-economic, perceptual and geographical.

## Declarations

### Author contribution statement

Wadii Snaibi: Conceived and designed the experiments; Performed the experiments; Analyzed and interpreted the data; Contributed reagents, materials, analysis tools or data; Wrote the paper.

Abdelhamid Mezrhab & Oumar Sy: Conceived and designed the experiments; Analyzed and interpreted the data; Wrote the paper.

John F. Morton: Analyzed and interpreted the data; Wrote the paper.

### Funding statement

John Morton's contribution was partially funded under the UK North Africa Technical Assistance Facility by arrangement with the British Embassy Rabat.

### Data availability statement

The data that has been used is confidential.

### Declaration of interests statement

The authors declare no conflict of interest.

### Additional information

No additional information is available for this paper.

## Appendix A Supplementary data

Table S.1 Meteorological stations_V2 Table S.1 Meteorological stations_V2

Table S.2 Explanatory variables_V2 Table S.2 Explanatory variables_V2

Table S.3 Factors influencing adaptation_V2 Table S.3 Factors influencing adaptation_V2
