# Supplementary material for: Perception and adaptation of pastoralists to climate variability and change in Morocco's arid rangelands
Source: Heliyon. 2021 Nov 23;7(11):e08434. doi: 10.1016/j.heliyon.2021.e08434 (PMC8640479; doi:10.1016/j.heliyon.2021.e08434)
Supplement: Table S.1 Meteorological stations_V2 [file mmc1.docx]

**Table S.1**

Meteorological stations, coordinates and periods of rainfall series

| Stations | Latitude | Longitude | Altitude (m) | Years |
| --- | --- | --- | --- | --- |
| Oujda | 34° 47’N | 1°56’W | 465 | 1914-2019 |
| Taourirt | 34°24’N | 02°53’W | 365 | 1923-2019 |
| Bni Mathar | 34°01’N | 02°02’W | 920 | 1931-2019 |
| Tendrara | 33°03’N | 02°00’W | 1460 | 1931-2019 |
| Bouaârfa | 32° 32’N | 01°58’W | 1200 | 1981-2019 |
| Figuig | 32°07’N | 01°14’W | 900 | 1935-2019 |
