# Supplementary material for: Perception and adaptation of pastoralists to climate variability and change in Morocco's arid rangelands
Source: Heliyon. 2021 Nov 23;7(11):e08434. doi: 10.1016/j.heliyon.2021.e08434 (PMC8640479; doi:10.1016/j.heliyon.2021.e08434)
Supplement: Table S.2 Explanatory variables_V2 [file mmc2.docx]

**Table S.2**

List and description of explanatory variables used in binary logistic regression analysis

| Variables | Type | Description and Measurement | Expected effect |
| --- | --- | --- | --- |
| Age | Continuous | Years | ± |
| Educational level | Dummy | 1: Literate and 0: Otherwise | + |
| Household size | Continuous | Numbers | + |
| Non-household labor force | Continuous | Numbers | + |
| Cultivated area | Continuous | Hectares | + |
| Sheep herd size | Continuous | Heads | + |
| Cattle herd size | Continuous | Heads | + |
| Agricultural or transport equipment | Continuous | Numbers | + |
| Access to formal credit | Dummy | 1: if access and 0: Otherwise | + |
| Training | Dummy | 1: Yes and 0: Otherwise | + |
| Membership in LPO | Dummy | 1: Yes and 0: Otherwise | + |
| Perceived temperature change | Dummy | 1: if perceived and 0: Otherwise | + |
| Perceived heavy rains | Dummy | 1: if perceived and 0: Otherwise | + |
| Perception of increased sandstorms | Dummy | 1: if perceived and 0: Otherwise | + |
| North agroecological site | Dummy | 1: North site and 0: Otherwise | + |
| Intermediate agroecological site | Dummy | 1: Intermediate site and 0: Otherwise | ± |

LPO: Livestock producers’ organization
