# Supplementary material for: Perception and adaptation of pastoralists to climate variability and change in Morocco's arid rangelands
Source: Heliyon. 2021 Nov 23;7(11):e08434. doi: 10.1016/j.heliyon.2021.e08434 (PMC8640479; doi:10.1016/j.heliyon.2021.e08434)
Supplement: Table S.3 Factors influencing adaptation_V2 [file mmc3.docx]

**Table S.3**

Factors hypothesized to influence the decision to adapt to CC in the HPEM

| Variables |  |
| --- | --- |
| Frequency (Percentage) | |
| Education | 49 (29.3) |
| Credit access | 38 (22.8) |
| Training | 34 (20.4) |
| Membership in LPO | 43 (25.7) |
| Perceived temperature change | 149 (89.2) |
| Perceived heavy rains | 111 (66.5) |
| Perception of increased sandstorms | 145 (86.8) |
| North agroecological site | 30 (18.0) |
| Intermediate agroecological site | 82 (49.0) |
| Mean ± Standard deviation | |
| Age of household head | 52 ± 13.65 |
| Household size | 8 ± 3.68 |
| Non-household labor force | 2 ± 1.81 |
| Cultivated area | 31.22 ± 46.28 |
| Equipment | 2 ± 2.19 |
| Sheep herd size | 167 ± 215.66 |
| Cattle herd size | 3 ±5.07 |
